# Association between Residential Proximity to Fuel-Fired Power Plants and Hospitalization Rate for Respiratory Diseases

**DOI:** 10.1289/ehp.1104146

**Published:** 2012-02-27

**Authors:** Xiaopeng Liu, Lawrence Lessner, David O. Carpenter

**Affiliations:** 1 Laboratory for Earth Surface Processes, College of Urban and Environmental Sciences, Peking University, Beijing, China; 2 Institute for Health and the Environment, University at Albany, State University of New York, Rensselaer, New York, USA

**Keywords:** asthma, COPD, particulates, respiratory infection, SO_2_

## Abstract

Background: Air pollution is known to cause respiratory disease. Unlike motor vehicle sources, fuel-fired power plants are stationary.

Objective: Using hospitalization data, we examined whether living near a fuel-fired power plant increases the likelihood of hospitalization for respiratory disease.

Methods: Rates of hospitalization for asthma, acute respiratory infection (ARI), and chronic obstructive pulmonary disease (COPD) were estimated using hospitalization data for 1993–2008 from New York State in relation to data for residences near fuel-fired power plants. We also explored data for residential proximity to hazardous waste sites.

Results: After adjusting for age, sex, race, median household income, and rural/urban residence, there were significant 11%, 15%, and 17% increases in estimated rates of hospitalization for asthma, ARI, and COPD, respectively, among individuals > 10 years of age living in a ZIP code containing a fuel-fired power plant compared with one that had no power plant. Living in a ZIP code with a fuel-fired power plant was not significantly associated with hospitalization for asthma or ARI among children < 10 years of age. Living in a ZIP code with a hazardous waste site was associated with hospitalization for all outcomes in both age groups, and joint effect estimates were approximately additive for living in a ZIP code that contained a fuel-fired power plant and a hazardous waste site.

Conclusions: Our results are consistent with the hypothesis that exposure to air pollution from fuel-fired power plants and volatile compounds coming from hazardous waste sites increases the risk of hospitalization for respiratory diseases.

Power plants provide electricity to satisfy a variety of needs of society. Some power plants, such as hydroelectric-, nuclear-, wind-, and solar-powered plants, provide energy without air pollution, but fuel-fired power plants are the major source of electrical power in most countries. Unfortunately, fuel-fired power plants emit millions of tons of air pollutants each year (Environmental Integrity Project 2007; Krewitt et al. 1998). In addition to formation and release of carbon dioxide, which is a major concern with regard to climate change, fuel-fired power plants release sulfur dioxide (SO_2_), nitrogen oxides (NO_x_), particulate matter (PM), and polycyclic aromatic hydrocarbons (PAHs) and may also release volatile organic compounds (Schneider 2004). Although these air pollutants are also released by motor vehicles and other sources of fossil-fuel combustion, fossil-fuel power plants are localized sources of elevated air pollution.

Air pollutant exposures are associated with increased risks of respiratory (Atkinson et al. 2001) and cardiovascular (Ito et al. 2011; Zanobetti and Schwartz 2005) diseases, and possibly also central nervous system diseases (Perera et al. 2008) and pregnancy complications (O’Connor and Roy 2008). SO_2_ and NO_x_ are known triggers for asthma attacks (Dales et al. 2006; Samoli et al. 2011) and increase risk for lung and heart disease (Jerrett et al. 2009; Wang et al. 2009). Smargiassi et al. (2009) investigated rates of asthmatic episodes in relation to short-term variations in SO_2_ levels coming from petroleum refineries and found significant elevations in both emergency department visits and hospitalizations. Ostro et al. (2009) reported daily rates of hospitalization of children for respiratory disease in relation to elevations in levels of air pollutants and found significantly elevated rates of hospitalization of 4.1% for PM ≤ 2.5 μm in aerodynamic diameter (PM_2.5_), 3.4% for organic carbon, and 3.3% for NO_x_. PM coming from power plants may contain PAHs and heavy metals (O’Connor and Roy 2008; Yager et al. 1997), and these exposures may contribute to other adverse health effects, including cancer (Pope et al. 2002) and neurotoxic effects (Edwards et al. 2010).

There is convincing evidence that exposure to air pollutants increases the risk of infectious respiratory disease. Chauhan and Johnston (2003) reviewed relationships between air pollution and respiratory infection and concluded that the association is strong and consistent, particularly for NO_x_. Children living in a community with high levels of PM with strong acidity (52 nmol/m^3^) were significantly more likely [odds ratio (OR) = 1.66; 95% confidence interval (CI): 1.11, 2.48] to report at least one episode of bronchitis in the past year compared with children living in the range of average city-specific air pollution (31.1 nmol/m^3^). Elevated fine particulate sulfate (6.8 μg/m^3^) was associated with higher rates of bronchitis (OR = 1.65; 95% CI: 1.12, 2.42) than were concentrations of 4.7 μg/m^3^ (Dockery et al. 1996).

Hermann et al. (2004) predicted that if 29 proposed fossil-fuel power plants in Virginia were operated for 6 years, PM_2.5_ in 272 counties would increase by > 0.01 μg/m^3^ and 104 additional premature deaths would occur. In a recent assessment, New York State was ranked third highest in incidence of adverse impacts attributable to power plant emissions (Schneider and Banks 2010). To our knowledge, no study has specifically documented elevations in disease in relation to residential proximity to power plants. It is reasonable to assume that living near a fuel-fired power plant poses a risk of exposure to combustion products or byproducts, such as SO_2_, NO_x_, PM_2.5_, and PAHs, and that these contaminants will be inhaled and have adverse effects on health.

The primary objective of this study was to assess the association of residential proximity to fuel-fired power plants with rates of hospitalization for asthma and infectious and chronic respiratory diseases. In addition, we explored combined effects with residential proximity to hazardous waste sites, which has previously been associated with asthma and infectious respiratory disease hospitalization (Carpenter et al. 2008; Ma et al. 2007).

## Methods

*Study population.* We used the New York Statewide Planning and Research Cooperative System (SPARCS) to obtain data on hospital discharges for the years 1993–2008. SPARCS is an administrative database maintained by the New York State Department of Health (NYSDOH; Albany, NY). For each inpatient upon discharge, every state-regulated hospital (except federal hospitals such as those operated by the Veterans Administration) must report to NYSDOH the primary diagnosis, and up to 14 other diagnoses, based on the *International Classification of Disease, Ninth Revision, Clinical Modification* (ICD-9-CM; National Center for Health Statistics 2006). In this study, we selected hospital discharge data that had diagnosis of *a*) asthma (ICD-9-CM code 493), *b*) acute respiratory infection (ARI; ICD-9-CM codes 460–466), and/or *c*) chronic obstructive pulmonary disease (COPD; ICD-9-CM codes 490–492 and 494–496) as either the primary or secondary diagnosis.

The SPARCS data used included patient age, sex, race/ethnicity, and ZIP code of current residence. New York City was excluded from this analysis because it maintains its own hospitalization listing, which is separate from that maintained by NYSDOH. New York City also differs significantly in population density and composition and in social and economic status. There are approximately 2.5 million hospitalizations per year in New York State other than in New York City.

This and other studies using the SPARCS data set have been reviewed by the institutional review board of the University at Albany and declared to be exempt because the data set does not include any unique identifiers.

*Demographic data.* Our unit of analysis was subpopulations within ZIP codes. Demographic data on New York State residents [age, sex, race/ethnicity, median household income (MHI), and population density for each ZIP code in New York State] was obtained from CLARITAS Inc. (http://www.claritas.com/sitereports/), a resource company that provides information derived from the 1990 and 2000 U.S. Census (Huang et al. 2006). In addition to excluding ZIP codes for New York City, we excluded 281 ZIP codes that included post boxes only or that had changed during 1993–2008, leaving 1,403 ZIP codes for analysis.

We estimated person-years based on the number of residents in each eligible ZIP code during each year for 1993–2008. The analysis was restricted to Caucasians (white) and African Americans (black) because these groups account for > 95% of total hospitalizations. Age was divided into eight groups (0, 1–2, 3–5, 6–9, 10–24, 25–49, 50–74, and ≥ 75 years) or dichotomized as < 10 or ≥ 10 years. To control for population density, ZIP codes with ≥ 1,000 persons/mi^2^ were classified as urban, and those with < 1,000 persons/mi^2^ were classified as rural.

*Exposure assessment.* We obtained a list of existing, operating power plants and electric generators in New York State for the years 1990–2008, including information on location, energy source, and operating period, from the U.S. Energy Information Administration (2010). Using this information, we identified ZIP codes that contained fuel-fired generators in all of New York State except for New York City. Preliminary analyses of hospitalization rates associated with residence in a ZIP code with a power plant classified according to the type of fuel used as an energy source (coal, oil, natural gas, landfill gas, and/or solid waste) did not indicate clear or consistent patterns [see Supplemental Material, Table S1 (http://dx.doi.org/10.1289/ehp.1104146)]. Therefore, we classified patients as exposed if they resided in a ZIP code with at least one fuel-fired power plant, regardless of the fuel used, at the time of hospitalization.

Previous studies reported elevated rates of hospitalization for asthma and infectious respiratory diseases among people living near contaminated waste sites (Carpenter et al. 2008; Ma et al. 2007). To mitigate confounding and identify interactions, we classified ZIP codes into four mutually exclusive groups: “fuel-only” ZIP codes contained one or more generators using coal, oil, natural gas, landfill gas, or solid waste as an energy source during the period of 1990–2008 but did not contain a hazardous waste site (*n* = 38); “waste-only” ZIP codes did not contain a fuel-fired power plant but did have a National Priority List site or state Superfund site, including ZIP codes that abutted bodies of water known to be contaminated with persistent organic pollutants (the lower Hudson River and six “areas of concern”; *n* = 347) (Carpenter et al. 2001); “fuel-and-waste” ZIP codes contained fuel-fired power plant(s) and hazardous waste site(s) (*n* = 84); and the remaining “clean” ZIP codes did not contain a fuel-fired power plant or a hazardous waste site (*n* = 934). Although hospitalization rates for the different diseases have changed over time (with asthma and COPD tending to increase, and ARIs tending to decrease), trends did not appear to differ by exposure status [see Supplemental Material, Figure S1 (http://dx.doi.org/10.1289/ehp.1104146)]. Therefore, we did not consider year-by-year data in this study.

We used MHI as a proxy measure of socioeconomic status (SES), another potential confounder in our study. Although there were no significant differences in mean MHI among the four classes of ZIP codes, their distributions varied [See Supplemental Material, Figure S2 (http://dx.doi.org/10.1289/ehp.1104146)]. In addition, preliminary analyses indicated substantial differences in crude hospitalization rates for ZIP codes with very high or very low MHI [See Supplemental Material, Table S2 (http://dx.doi.org/10.1289/ehp.1104146)]. Therefore, we restricted the analysis to ZIP codes with year 2000 MHI ranging from $23,057.4 (the 5th percentile of MHI) to $71,506.6 (the 95th percentile of MHI), leaving 841 clean, 32 fuel-only, 301 waste-only, and 78 fuel-and-waste ZIP codes for analysis (total *n* = 1,252). MHI in the remaining ZIP codes was categorized into quartiles: $23,057.4–31,069.3, $31,069.3–36,179.9, $36,179.9–44,204.4, and $44,204.4–71,506.6.

*Statistical analysis.* We calculated hospitalization rates per 100,000 person-years as the number of discharge diagnoses for each outcome of interest divided by the total person-years for the population residing in each ZIP code category for the years 1993–2008. Rate ratios (RRs) of hospitalization and their 95% CIs were calculated for fuel-only, waste-only, and fuel-and-waste ZIP codes, with clean ZIP codes as the common referent exposure group.

Covariate patterns were formed that controlled for age, sex, race/ethnicity, MHI, and exposure. The outcome for each covariate pattern was the number of hospital discharge diagnoses across all the ZIP codes having that covariate pattern. We used separate negative binomial, log-linear regression models to estimate associations between exposure [modeled as categorical indicator variables for fuel-only, waste-only, or fuel-and-waste vs. clean ZIP codes (referent)], and hospitalization rates among those < 10 or ≥ 10 years of age, with adjustment for potential confounders. For children < 10 years of age, we estimated associations between exposure and hospitalization rates for asthma or ARI using separate models adjusted for sex, race/ethnicity (black or white), age (categorical: 1–2, 3–5, or 6–9 vs. 0 years), MHI (categorical: first-, second-, or third-quartile MHI vs. fourth-quartile MHI), and urban/rural ZIP code. For those ≥ 10 years of age, we estimated associations with hospitalization for asthma, ARI, or COPD using separate models adjusted for the same covariates but with age categorized as 25–49, 50–74, or ≥ 75 versus 10–24 years.

All statistical analyses were performed with SAS software (version 9.2; SAS Institute Inc., Cary, NC, USA). *p*-Value < 0.05 was selected as the critical value for statistical significance for all statistical tests. The PROC GENMOD procedure was used for negative binomial regression analysis (Huang et al. 2006; Shcherbatykh et al. 2005).

## Results

During 1993–2008, there were 90,773 asthma and 98,298 ARI hospitalizations for children < 10 years of age, and 820,671 asthma, 233,171 ARI, and 1,948,903 COPD hospitalizations among those ≥ 10 years of age, in the 1,252 ZIP codes included in the analysis (Table 1). Although African Americans contributed only 8.7% of the person-years studied, they accounted for 17.8% of asthma and 13.7% of ARI hospitalizations.

**Table 1:** Demographic characteristics and hospitalizations of the studied population [n (%)].

Hospitalizations				
Characteristic	Person-yearsa	Asthma	ARI	COPD
Total population		164,289,936 (100.0)		911,444 (100.0)		331,469 (100.0)		1,948,903 (100.0)
Exposure								
Clean		65,271,804 (39.7)		320,693 (35.2)		118,239 (35.7)		692,247 (35.5)
Fuel only		4,529,951 (2.8)		24,765 (2.7)		8,466 (2.6)		55,126 (2.8)
Waste only		69,479,535 (42.3)		403,334 (44.3)		144,613 (43.6)		848,798 (43.6)
Fuel and waste		25,008,646 (15.2)		162,652 (17.8)		60,151 (18.1)		352,732 (18.1)
Sex								
Female		84,417,102 (51.4)		600,327 (65.9)		179,061 (54.0)		995,977 (51.1)
Male		79,872,834 (48.6)		311,117 (34.1)		152,408 (46.0)		952,926 (48.9)
Race								
African American		14,339,797 (8.7)		162,482 (17.8)		45,357 (13.7)		100,906 (5.2)
Caucasian		149,950,139 (91.3)		748,962 (82.2)		286,112 (86.3)		1,847,997 (94.8)
Age group (years)								
0		2,057,008 (1.3)		13,340 (1.5)		55,502 (16.7)		446 (0.0)
1–2		4,263,638 (2.6)		31,052 (3.4)		26,302 (7.9)		520 (0.0)
3–5		6,456,134 (3.9)		25,724 (2.8)		10,505 (3.2)		392 (0.0)
6–9		8,747,610 (5.3)		20,657 (2.3)		5,989 (1.8)		349 (0.0)
10–24		33,073,852 (20.1)		96,648 (10.6)		24,247 (7.3)		4,447 (0.2)
25–49		58,830,783 (35.8)		297,430 (32.6)		62,607 (18.9)		98,675 (5.1)
50–74		39,683,599 (24.2)		290,371 (31.9)		74,766 (22.6)		928,603 (47.6)
≥ 75		11,177,312 (6.8)		136,222 (14.9)		71,551 (21.6)		915,471 (47.0)
aPerson-years determined by the numbers of residents in the study ZIP codes during each year (1993–2008) according to characteristic.								

Crude hospitalization rates in both fuel-only and waste-only ZIP codes were significantly higher than those in clean ZIP codes for all outcomes with the exception of rates of asthma and ARI in fuel-only ZIP codes among those < 10 years of age (Table 2). Crude discharge rates for all outcomes were higher in ZIP codes with a fuel-fired power plant and a hazardous waste site than in ZIP codes with only a fuel or waste site. In general, these associations were maintained when estimates were stratified according to age (< 10 or ≥ 10 years) and sex and race/ethnicity [see Supplemental Material, Table S3 (http://dx.doi.org/10.1289/ehp.1104146)].

**Table 2:** Crude hospital discharge rates for asthma, ARI, and COPD according to age and exposure after excluding extremes of MHI status.

Person-years	Hospital discharge rate per 100,000 (95% CI)				
	Exposure	Asthma	ARI	COPD
Age < 10 years								
Clean		8,661,904		359 (355, 363)		404 (400, 409)		
Fuel only		567,857		381 (365, 397)		414 (397, 431)		
Waste only		8,939,610		452 (448, 457)		474 (470, 479)		
Fuel and waste		3,355,019		509 (501, 517)		551 (544, 559)		
Age ≥ 10 years								
Clean		56,609,900		512 (510, 513)		147 (146, 148)		1,222 (1,219, 1,225)
Fuel only		3,962,094		570 (563, 578)		154 (151, 158)		1,390 (1,379, 1,402)
Waste only		60,539,925		599 (597, 601)		169 (168, 170)		1,401 (1,398, 1,404)
Fuel and waste		21,653,627		672 (669, 676)		192 (191, 194)		1,627 (1,622, 1,633)

After adjusting for known confounders (sex, race/ethnicity, age, MHI, urban/rural), living in a fuel-only ZIP code was associated with slightly elevated (not significant) rates of asthma and ARI hospitalization compared with living in a clean ZIP code (Table 3). Residence in a waste-only ZIP code, however, was associated with significantly increased RRs for hospitalization for asthma (RR = 1.11; 95% CI: 1.03, 1.19) and ARI (RR = 1.13; 95% CI 1.05, 1.21) relative to clean ZIP codes. Stronger associations were observed with residence in a fuel-and-waste ZIP code (respectively, RR = 1.19; 95% CI: 1.11, 1.28; and RR = 1.24; 95% CI: 1.15, 1.33).

**Table 3:** Adjusted RRs of hospital discharge for asthma, ARI, and COPD as a function of residence in ZIP codes with different exposure status.

Asthma							ARI							COPD		
Age < 10 years			Age ≥ 10 years			Age < 10 years			Age ≥ 10 years			Age ≥ 10 years		
Exposure	RR (95% CI)	p-Value	RR (95% CI)	p-Value	RR (95% CI)	p-Value	RR (95% CI)	p-Value	RR (95% CI)	p-Value
Clean		1.00				1.00				1.00				1.00				1.00		
Fuel only		1.01 (0.91, 1.12)		0.85		1.11 (1.02, 1.20)		0.01		1.03 (0.93, 1.14)		0.56		1.15 (1.05, 1.27)		0.003		1.17 (1.06, 1.29)		0.002
Waste only		1.11 (1.03, 1.19)		0.005		1.07 (1.00, 1.14)		0.04		1.13 (1.05, 1.21)		0.001		1.09 (1.02, 1.17)		0.01		1.16 (1.08, 1.26)		0.0001
Fuel and waste		1.19 (1.11, 1.28)		< 0.0001		1.18 (1.11, 1.26)		< 0.0001		1.24 (1.15, 1.33)		< 0.0001		1.21 (1.13, 1.30)		< 0.0001		1.26 (1.17, 1.37)		< 0.0001


Among those ≥ 10 years of age, RRs were significantly elevated among residents in fuel-only ZIP codes compared with clean ZIP codes for asthma (RR = 1.11; 95% CI: 1.02, 1.20), ARI (RR = 1.15; 95% CI: 1.05, 1.27), and COPD (RR = 1.17; 95% CI: 1.06, 1.29) and slightly higher than corresponding RRs for waste-only ZIP codes (Table 3). The largest RRs estimated for all outcomes were for fuel-and-waste ZIP codes. The RRs for residence in a ZIP code with both exposures were consistent with expectations assuming additive effects of residence in fuel-only and waste-only ZIP codes. Supplemental Material, Table S4 (http://dx.doi.org/10.1289/ehp.1104146), shows the relation of hospital discharge for asthma, ARI, and COPD with covariates, sex, race/ethnicity, age, income, and urban/rural residence for those < 10 and ≥ 10 years of age.

Hospitalization rates in all interaction subgroups with sex, race/ethnicity, age, MHI, and urban/rural status were compared to identify effect modifiers (data not shown). There was no statistically significant effect on the RR or improvement in the overall quality of fit (deviance/degree of freedom) upon consideration of most interaction subgroups, and we did not see any evidence of significant interactions (*p* > 0.05 based on deviance statistics).

## Discussion

There is mounting evidence that environmental exposures increase the risk of human disease, and these exposures come from multiple sources, including air pollutants coming from fossil-fuel combustion and hazardous chemicals found in waste sites and many other places. Although many of the important sources of air pollutants are mobile, power plants, because they are stationary, constitute a localized source of air toxics and thus allow one to examine whether individuals living near these power plants experience greater exposure and more disease as a result. The goal of this study was to examine rates of hospitalization for asthma and respiratory infection among individuals who live near fuel power plants. Because previous studies from our group have reported elevated rates of hospitalization for these same diseases among individuals living near hazardous waste sites (Carpenter et al. 2008; Ma et al. 2007), we have reexamined these relationships now with more years of data to assess the rate of hospitalization among individuals who live near both a fuel-fired power plant and a hazardous waste site.

*Interactions between proximity of residence to fuel-fired power plants and waste sites.* We found a significant elevation in rates of hospitalization for respiratory diseases among individuals > 10 years of age who lived near a fuel-fired power plant after adjusting for age, sex, race/ethnicity, MHI, and urban/rural residence. We confirmed that living near waste sites was associated with significantly increased rates of hospitalization for asthma, ARI, and COPD.

The results of the present study show that hospitalization rates in individuals living in ZIP codes with both a fuel-fired power plant and a hazardous waste site are increased compared with rates associated with exposure to either factor alone, and the RRs are approximately additive. This indicates how important it is in a study such as this to control for both types of exposure. Although asthma and ARI hospitalizations in children < 10 years of age did not show significant associations with residence near fuel-fired power plant sites, residence near both a fuel-fired power plant and a hazardous waste site was associated with higher rates of hospitalization than residence near a waste site alone.

*Limitation or bias.* There are several methodical limitations in this study. Residence in a ZIP code containing a fuel-fired power plant or a waste site was our only measure of exposure. Because fuel-fired power plants usually have high stacks, the contaminants may migrate beyond the geographic area covered by a ZIP code. Thus, residents in a “clean” ZIP code could be exposed if they are downwind of a fuel-fired power plant (Pala et al. 2010). We do not have information on the height of the stacks of individual power plants or their location within ZIP codes, and we did not control for wind direction or strength. We did not have data available to control for potential confounders such as genetic predisposition, smoking, diet, and other personal habits. We monitored only hospitalizations and did not have information about emergency department or physician office visits. We also cannot distinguish multiple hospitalizations for one individual from those for several individuals, and we did not have information on residence locations at times other than at hospitalization. New York State residents who sought out-of-state health care were not included in this data set, nor were patients admitted to federally regulated hospitals. SES is a particularly important confounder that was only partially controlled for in our study through the use of MHI for each ZIP code.

*Strengths of the study.* There are, however, major strengths of this study. We had data on a very large population over a long period of time and could control for age, sex, race/ethnicity, urban/rural residence, and ZIP-code-level MHI as a surrogate for SES. In spite of the limitations, most of which would tend to obscure risk relationships, we found a significant elevation in rates of hospitalization for asthma, ARI, and COPD among individuals living in ZIP codes containing fuel-fired power plants and confirmed our previous findings that residence near hazardous waste sites is also associated with increased hospitalization rates for the outcomes examined. Moreover, our results are consistent with those of other investigations relating respiratory disease hospitalization to exposure to air pollutants (O’Connor and Roy 2008; Pala et al. 2010).

Although semiecologic studies such as ours must always be considered to be hypothesis generating, the congruence between these results and previous information provides additional evidence that use of the large SPARCS data set has value in identifying patterns of disease in relation to residential exposures.

## Supplemental Material

(2.5 MB) PDF
