# Supplementary material for: Association between Residential Proximity to Fuel-Fired Power Plants and Hospitalization Rate for Respiratory Diseases
Source: Environ Health Perspect. 2012 Feb 27;120(6):807–10. doi: 10.1289/ehp.1104146 (PMC3385425; doi:10.1289/ehp.1104146)
Supplement: (2.5 MB) PDF [file ehp.1104146.s001.pdf]

Supplemental Material

Association between Residential Proximity to Fuel-Fired Power Plants and Hospitalization Rate for Respiratory Diseases

Xiaopeng Liu [1, 2], Lawrence Lessner [2] and David O. Carpenter [2, 3]

[1] Laboratory for Earth Surface Processes

College of Urban and Environmental Sciences

Peking University

Beijing 100871, China

[2] Institute for Health and the Environment

University at Albany

East Campus, Room A 217

Rensselaer, NY 12144

[3] Correspondence to:

David O. Carpenter, M.D.

Institute for Health and the Environment

University at Albany

5 University Place

Rensselaer, NY 12144

518-525-2660

FAX 518-525-2665

dcarpenter@albany.edu

Table S1. Crude hospital discharge rates for zip codes with fuel-fired power plants according to fuel type.

Age	Fuel type (No. of zip codes)	Hospital discharge rate, per 100,000 (95% CI)		
		Asthma	ARI	COPD
Age <10	Coal (10)	464 (438, 491)	528 (501, 557)	
	Gas (41)	532 (520, 544)	590 (577, 602)	
	LFG (19)	269 (254, 285)	310 (294, 327)	
	Oil (14)	233 (210, 259)	248 (225, 275)	
	Solid (11)	451 (432, 471)	455 (436, 475)	
	Coal+Gas (1)	73 (25, 214)	316 (185, 541)	
	Coal+Oil (2)	660 (585, 744)	551 (484, 628)	
	Gas+Oil (17)	527 (510, 545)	560 (542, 578)	
	Gas+Solid (1)	513 (459, 573)	794 (726, 868)	
	LFG+Oil (2)	292 (226, 377)	347 (274, 439)	
	Coal+Gas+Oil (3)	820 (781, 861)	870 (830, 912)	
	Gas+Oil+Solid (1)	500 (454, 552)	443 (399, 492)	
Age ≥10	Coal (10)	602 (590, 614)	184 (178, 191)	1,483 (1,464, 1,503)
	Gas (41)	723 (718, 729)	212 (209, 215)	1,852 (1,843, 1,861)
	LFG (19)	486 (478, 494)	141 (137, 145)	1,303 (1,290, 1,316)
	Oil (14)	438 (426, 450)	135 (129, 142)	1,248 (1,228, 1,268)
	Solid (11)	657 (648, 666)	181 (177, 186)	1,427 (1,413, 1,440)

[Table S1 Continued]

Age	Fuel type (No. of zip codes)	Hospital discharge rate, per 100,000 (95% CI)		
		Asthma	ARI	COPD
	Coal+Gas (1)	220 (169, 287)	80 (52, 124)	761 (660, 877)
	Coal+Oil (2)	447 (424, 471)	162 (148, 177)	1,235 (1,196, 1,274)
	Gas+Oil (17)	713 (705, 721)	182 (178, 186)	1,432 (1,421, 1,444)
	Gas+Solid (1)	690 (663, 719)	206 (191, 222)	2,325 (2,274, 2,377)
	LFG+Oil (2)	951 (898, 1,008)	186 (164, 212)	2,345 (2,260, 2,433)
	Coal+Gas+Oil (3)	778 (762, 793)	243 (235, 252)	1,586 (1,564, 1,609)
	Gas+Oil+Solid (1)	568 (547, 589)	129 (120, 139)	1,355 (1,323, 1,387)

Table S2. Crude comparison on hospital discharge rates between selected zip codes and excluded zip codes according to income[a].

Age	MHI	Hospital discharge rate, per 100,000 (95% CI)		
		Asthma	ARI	COPD
Age <10	< 23,057[b]	804 (783, 825)	670 (651, 690)	
	23,057-71,507[c]	435 (432, 438)	480 (477, 484)	
	> 71,507[d]	249 (243, 255)	260 (254, 266)	
Age ≥10	< 23,057[b]	1,106 (1,095, 1,116)	275 (270, 281)	1,695 (1,682, 1,708)
	23,057-71,507[c]	586 (585, 588)	169 (168, 170)	1,430 (1,428, 1,433)
	> 71,507[d]	412 (409, 414)	110 (109, 112)	927 (923, 931)

a. – all further analysis excluded zip codes with MHI <23,057 and >71,507

b. – including 118 zip codes: 81 clean, 2 fuel only, 31 waste only and 4 fuel and waste.

c. – including 1252 zip codes: 841 clean, 32 fuel only, 301 waste only and 78 fuel and waste.

d. – including 33 zip codes: 12 clean, 4 fuel only, 15 waste only and 2 fuel and waste.

Table S3. Stratified comparison of unadjusted hospital discharge rates for asthma, ARI and COPD by age, race/ethnicity and sex.

Age	Subpopulation	Exposure	Hospital discharge rate, per 100,000 (95% CI)		
			Asthma	ARI	COPD
Age <10	White-Female	Clean[a]	232 (227, 236)	315 (309, 320)	
		Fuel Only[b]	221 (203, 240)	305 (284, 328)	
		Waste Only[c]	267 (262, 272)	360 (354, 366)	
		Fuel and Waste[d]	317 (308, 327)	430 (419, 441)	
	White-Male	Clean	403 (397, 409)	465 (458, 471)	
		Fuel only	373 (350, 398)	445 (420, 472)	
		Waste only	471 (464, 478)	515 (508, 522)	
		Fuel and Waste	528 (516, 539)	595 (582, 607)	
	Black-Female	Clean	586 (561, 611)	454 (433, 477)	
		Fuel only	810 (716, 915)	568 (491, 658)	

[Table S3. Continued]

Age	Subpopulation	Exposure	Hospital discharge rate, per 100,000 (95% CI)		
			Asthma	ARI	COPD
		Waste only	760 (738, 783)	603 (583, 623)	
		Fuel and Waste	766 (731, 803)	657 (624, 692)	
	Black-Male	Clean	985 (953, 1,017)	635 (610, 661)	
		Fuel only	1,229 (1,115, 1,355)	827 (734, 931)	
		Waste only	1,198 (1,171, 1,226)	801 (779, 824)	
		Fuel and Waste	1,317 (1,271, 1,364)	919 (881, 959)	
Age ≥10	White-Female	Clean	629 (626, 632)	164 (162, 165)	1,224 (1,220, 1,229)
		Fuel only	678 (667, 690)	170 (164, 176)	1,422 (1,405, 1,439)
		Waste only	714 (711, 717)	184 (182, 186)	1,435 (1,430, 1,439)
		Fuel and Waste	818 (812, 823)	215 (212, 218)	1,725 (1,717, 1,733)
	White-Male	Clean	315 (313, 317)	120 (119, 122)	1,302 (1,298, 1,306)
		Fuel only	345 (336, 353)	126 (121, 131)	1,459 (1,441, 1,477)
		Waste only	336 (334, 339)	133 (132, 134)	1,462 (1,457, 1,466)
		Fuel and Waste	373 (369, 377)	150 (147, 152)	1,681 (1,673, 1,689)

[Table S3. Continued]

Age	Subpopulation	Exposure	Hospital discharge rate, per 100,000 (95% CI)		
			Asthma	ARI	COPD
		Fuel only	1,592 (1,533, 1,653)	257 (234, 282)	776 (735, 819)
		Waste only	1,668 (1,653, 1,683)	299 (293, 305)	910 (899, 921)
		Fuel and Waste	1,769 (1,744, 1,795)	319 (308, 330)	902 (884, 920)
	Black-Male	Clean	632 (621, 644)	164 (158, 169)	678 (667, 690)
		Fuel only	675 (635, 718)	176 (156, 199)	894 (848, 942)
		Waste only	768 (757, 778)	214 (209, 220)	997 (985, 1,009)
		Fuel and Waste	852 (834, 870)	229 (220, 238)	885 (867, 904)

a. Clean – zip code containing neither a fuel-fired power plant nor a hazardous waste site;

b. Fuel only – zip code containing a fuel-fired power plant, without a waste site;

c. Waste only – zip code containing a hazardous waste site, without a fuel-fired power plant;

d. Fuel and waste – zip code containing both a fuel-fired power plant and a hazardous waste site.

Table S4. Adjusted rate ratios of hospital discharge in relation to gender, race/ethnicity, age, MHI and urban/rural residence for individuals <10 and ≥10 years of age.

Parameter	*Asthma*				*ARI*				*COPD*	
	**< 10 years old**		**≥ 10 years old**		**< 10 years old**		**≥ 10 years old**		**≥ 10 years old**	
	**RR (95% CI)**	***p*-Value**	**RR (95% CI)**	***p*-Value**	**RR (95% CI)**	***p*-Value**	**RR (95% CI)**	***p*-Value**	**RR (95% CI)**	***p*-Value**
**Gender**										
**Female**	1.00		1.88 (1.79, 1.97)	<0.0001	1.00		1.28 (1.22, 1.36)	<0.0001	1.00	<0.0001
**Male**	1.68 (1.59, 1.77)	<.0001	1.00		1.40 (1.33, 1.48)	<0.0001	1.00		1.21 (1.14, 1.28)	
**Race**										
**African American**	2.31 (2.19, 2.44)	<0.0001	2.16 (2.06, 2.67)	<0.0001	1.43 (1.35, 1.51)	<0.0001	1.694 (1.60, 1.79)	<0.0001	1.05 (0.99, 1.12)	0.09
**Caucasian**	1.00		1.00		1.00		1.00		1.00	
**Age**										
**0**	1.00				1.00					
**1-2**	1.14 (1.06, 1.24)	0.0007			0.23 (0.22, 0.25)	<0.0001				
**3-5**	0.66 (0.61, 0.71)	<0.0001			0.06 (0.06, 0.07)	<0.0001				
**6-9**	0.40 (0.37, 0.43)	<0.0001			0.03 (0.02, 0.03)	<0.0001				
**10-24**			1.00				1.00		1.00	

[Table S4. Continued]

Parameter	*Asthma*				*ARI*				*COPD*	
	< 10 years old		≥ 10 years old		< 10 years old		≥ 10 years old		≥ 10 years old	
**Age**	**RR (95% CI)**	***p*-Value**	**RR (95% CI)**	***p*-Value**	**RR (95% CI)**	***p*-Value**	**RR (95% CI)**	***p*-Value**	**RR (95% CI)**	***p*-Value**
**50-74**			2.69 (2.52, 2.88)	<0.0001			2.83 (2.62, 3.06)	<0.0001	172.07 (156.71, 188.92)	<0.0001
**≥ 75**			3.87 (3.62, 4.15)	<0.0001			7.79 (7.20, 8.42)	<0.0001	582.72 (530.44, 640.08)	<0.0001
**Income**										
**1Q MHI**	1.42 (1.31, 1.53)	<0.0001	1.43 (1.34, 1.52)	<0.0001	1.55 (1.44, 1.67)	<0.0001	1.59 (1.47, 1.71)	<0.0001	1.84 (1.69, 1.99)	<0.0001
**2Q MHI**	1.14 (1.06, 1.23)	0.001	1.21 (1.14, 1.30)	<0.0001	1.25 (1.16, 1.35)	<0.0001	1.43 (1.32, 1.54)	<0.0001	1.38 (1.27, 1.50)	<0.0001
**3Q MHI**	1.12 (1.04, 1.21)	0.004	1.18 (1.10, 1.26)	<0.0001	1.26 (1.17, 1.36)	<0.0001	1.32 (1.22, 1.42)	<0.0001	1.29 (1.19, 1.40)	<0.0001
**4Q MHI**	1.00		1.00		1.00		1.00		1.00	
**Urban/ Rural**										
**Urban**	1.07 (1.01, 1.13)	0.02	1.16 (1.11, 1.22)	<0.0001	0.95 (0.90, 1.01)	0.07	1.00 (0.94, 1.05)	0.87	0.95 (0.89, 1.01)	0.07
**Rural**	1.00		1.00		1.00		1.00		1.00	

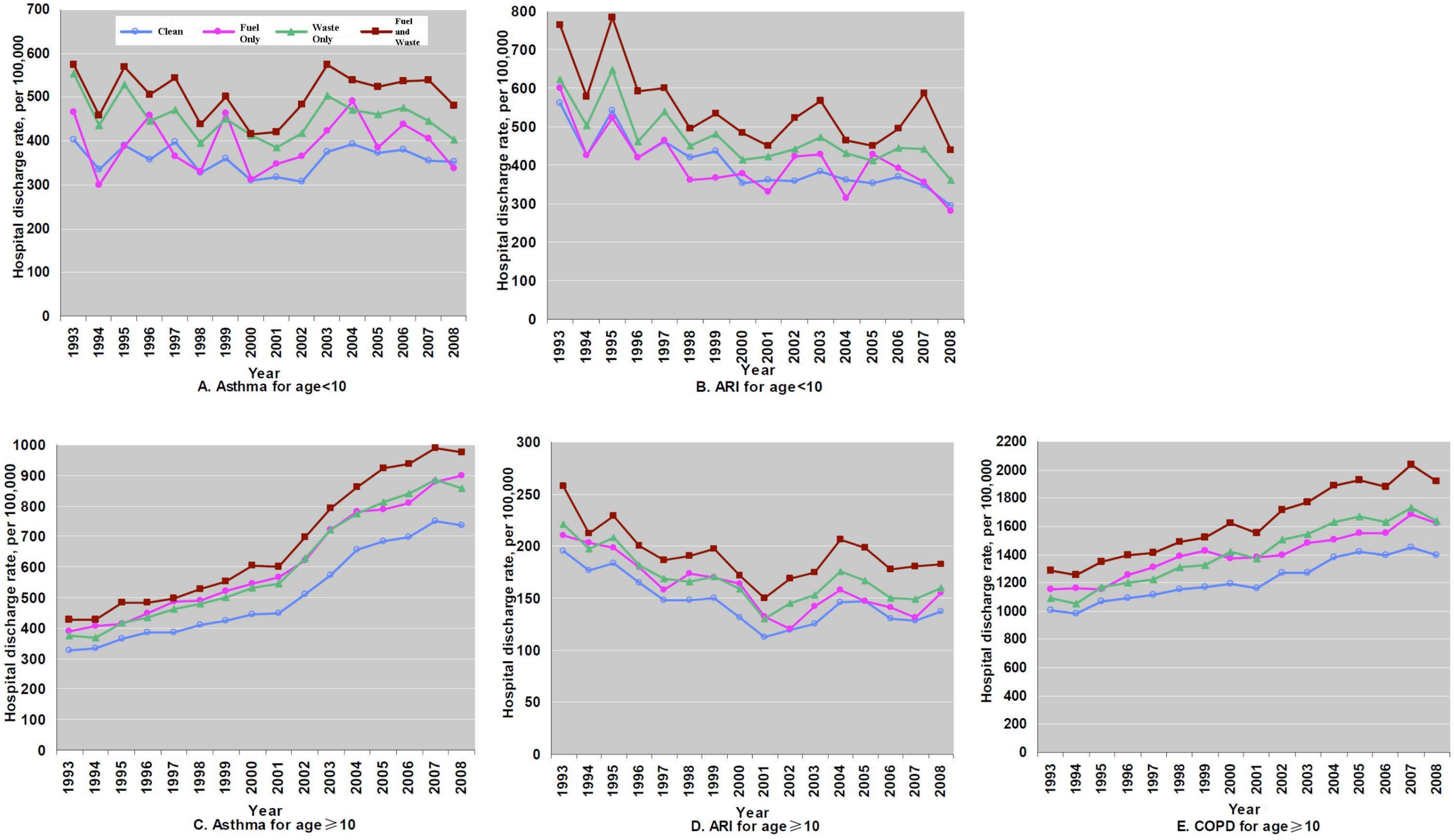

Figure S1. Time-series analysis on annual hospital discharge rate in 1993-2008 according to exposure.

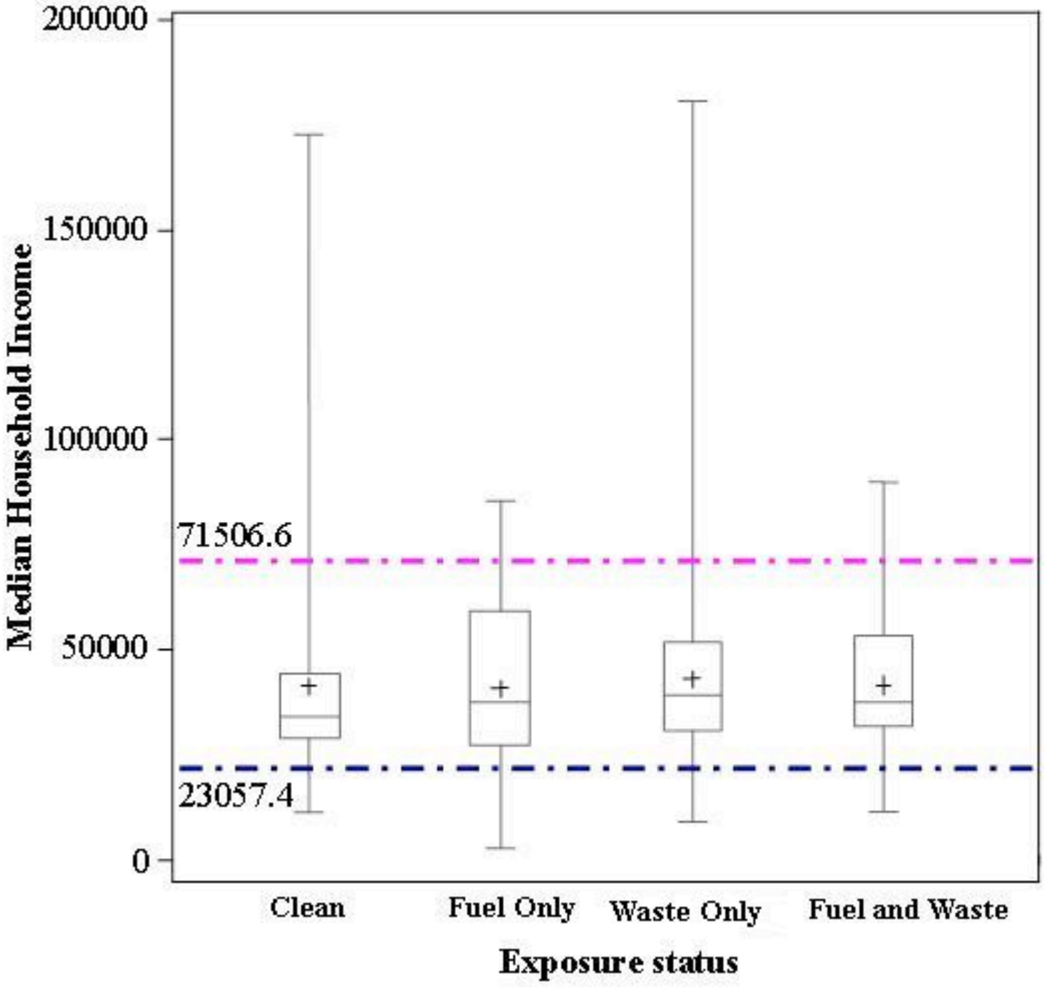

Figure S2. Comparison of MHIs according to exposure, indicating that the distribution of MHI in zip codes containing a fuel-fired power plant is not as dispersive as others.
